# Explanation for symptoms and biographical repair in a clinic for persistent physical symptoms

**DOI:** 10.1016/j.ssmqr.2024.100438

**Published:** 2024-06

**Authors:** Tom Sanders, Kate Fryer, Monica Greco, Cara Mooney, Vincent Deary, Christopher Burton

**Affiliations:** a Department of Social Work, Education and Community Wellbeing, Northumbria University, Northumbria Building, Newcastle upon Tyne, NE1 8ST, UK; b Division of Population Health, Sam Fox House, Northern General Hospital, University of Sheffield, Sheffield, S5 7AU, UK; c Department of Social and Policy Sciences, University of Bath, Claverton Down, Bath, BA2 7AY; d Clinical Trials Research Unit, School for Health & Related Research, University of Sheffield, Innovation Centre, Sheffield, S1 4DA, UK; e Department of Psychology, Northumbria University, Northumbria Building, Newcastle upon Tyne, NE1 8ST, UK

**Keywords:** Biographical repair, Persistent physical symptoms, Primary care, Biographical disruption, Symptoms clinic, Qualitative research

## Abstract

**Introduction:**

Biographical disruption describes the process by which illness impacts not just on a person's body and their participation in activities, but also on their sense of self. Biographical disruption is often followed by a process of biographical repair in which identity is reconstructed and a new normality is restored. People with persistent physical symptoms (sometimes referred to as medically unexplained symptoms) experience biographical disruption. This can be complicated by lack of explanation and the implication that if the problem is not medical, then it might be the person/psychological. We aimed to examine this tension in people attending a novel “Symptoms Clinic” for people with persistent physical symptoms.

**Methods:**

This study reports an embedded qualitative study in a UK based randomised controlled trial. Data were collected by audio recordings of consultations and semi-structured interviews with patients. We used theoretically informed thematic analysis with regular coding and discussion meetings of the analysis team. This analysis explores the role of intervention components in facilitating biographical repair.

**Results:**

The lack of acceptable explanation for persistent symptoms acted as a block to biographical repair. In the clinic, multi-layered explanations were offered and negotiated that viewed persistent symptoms as understandable entities rather than as indicators of something still hidden. These explanations allowed study participants to make sense of their symptoms and in turn opened new opportunities for self-management. The result was that participants were able to reframe their symptoms in a way that enabled them to see themselves differently. Even if symptoms had not yet improved, there was a sense of being better. This can be understood as a process of biographical repair.

**Conclusion:**

Explaining persistent physical symptoms enables biographical repair.

## Highlights

• Persistent physical symptoms lead to biographical disruption.
• Absence of an explanation for these symptoms blocks biographical repair.
• Extended role GPs co-produced multi-layered explanations for symptoms with patients.
• Explanations were acceptable and opened new opportunities for action and learning.
• Explanation of persistent symptoms facilitated biographical repair.

## 1 Introduction

### 1.1 Biographical disruption and repair

Chronic illness affects not just a person's body and their participation in ordinary activities, but also their sense of self (Charmaz, 1983). A widely used concept to examine the impact on the self is that of biographical disruption (Bury, 1982; Lawton, 2003). Drawing on the idea that narratives are key to the ways in which we make sense of experiences, the concept of biographical disruption points to how an event of illness can disrupt the biographical narrative that previously supported a coherent sense of self. Biographical disruption could include restricted capabilities, being discredited, and having to rely more on others (Bury, 1982). While the concept of biographical disruption is forty years old, it remains relevant (Locock, 2015) and has been applied to a number of situations including illness with gradual rather than abrupt onset (Williams, 2000). Alongside biographical disruption, there has grown a literature on biographical reconstruction (Williams, 1984) and repair (Kuluski et al., 2014; Locock et al., 2009) in which individuals seek to cope with the change in their life by reconstructing their identity and restoring a sense of normality (Kuluski et al., 2014).

Biographical disruption is not an inevitable feature of illness. Particularly in older age, illness can be viewed as a continuation, or “flow” rather than a disruption (Faircloth et al., 2004). In some instances, the changes in identity associated with illness can be represented as biographical reinforcement (Wells et al., 2023). When it does occur, biographical disruption may not be a discrete event; a study of people with Meniere's disease described dynamic patterns termed “biographical oscillation” (Bell et al., 2016) while people with sciatica have been described as experiencing “biographical suspension”, a liminal state between injury and recovery (Saunders et al., 2018). Other work has framed biographical disruption in terms of embodiment, as a feature of “the relationship between the bodily effects of illness and a subject's pre-existing embodied orientation towards the world, and of the considerations of identity that those effects give rise to” (Engman, 2019, p. 126).

In many illness accounts, diagnosis, the point at which the illness is named or revealed, is a crucial moment. A clinical diagnosis provides a tangible reality for people which can help them adapt to, and live with, illness (Charmaz, 1983). All illnesses have social representations (negative and positive), which are discourses through which people learn to understand the condition and symptoms and what these mean to them. These illness discourses enable individuals to understand and therefore redefine (reconstruct) their biography, helping them come to terms and manage their illness (Clarke & James, 2003; Weitz, 1989). When there is no diagnosis (or no acceptable diagnosis or explanation for symptoms) there may be no such discourses to support the management of illness, while negative cultural tropes (e.g. ‘faking it’) may come into play to add to the sense of a spoiled identity and disrupted biography (Bean et al., 2022; Rossen et al., 2019).

### 1.2 Persistent physical symptoms

“Persistent physical symptoms” is a recently introduced expression to describe ongoing physical symptoms that are disproportionate to demonstrable disease (either clinically or through diagnostic tests). Persistent physical symptoms can exist either on their own (e.g. palpitations or headache) or in clusters represented by so-called functional somatic disorders (Rosendal et al., 2017). While all medical specialties have their own syndromes – e.g. fibromyalgia in rheumatology; irritable bowel syndrome in gastroenterology – there are theoretical and pragmatic arguments for viewing persistent physical symptoms as overlapping, with shared common processes (Den Boeft et al., 2017). Although persistent physical symptoms have often been referred to as “medically unexplained symptoms”, there is increasing evidence that they can be understood and explained. This understanding is multi-layered, including neurological processes by which the brain senses, interprets and regulates the body (Chen et al., 2021; Henningsen et al., 2018), psychological and social processes by which personal experience, emotions and interpretation influence perceptions of and responses to the body (Deary, Chalder, & Sharpe, 2007; Van Den Bergh et al., 2017), and bodily processes such as inflammation or disordered function which may precede or follow from these other layers. Considering these multi-layered processes together permits an understanding of symptoms as entities in their own right (Wardrope & Reuber, 2022) in a way which is analogous to current understanding of chronic pain (Fitzcharles et al., 2021). Thus, persistent symptoms are less signifiers of a specific causal pathology, and more indicators of problems in the (multi-layered) systems for managing signifiers of pathology.

This step of considering persistent symptoms (and the related construct of “functional disorders”) as entities in their own right represents an important shift away from viewing symptoms as indicators of either disease or, if no disease can be found, something else such as mental distress (Burton, 2003; Burton, Fink, Henningsen, Lowe, & Rief, 2020; Löwe et al., 2022). Historical dichotomies about the contested nature of symptoms exist in various forms including physical/mental; organic/functional (Stone & Carson, 2017); or “medically explained”/“medically unexplained” (Creed et al., 2010). In this paper, we use the term persistent physical symptoms (Rosendal et al., 2017) for two reasons: first, because it is more acceptable to patients (Picariello et al., 2015; Marks & Hunter, 2015) and second, because explanation for persistent symptoms in terms of the body, brain and signalling between them is increasingly feasible (Burton, Lucassen, Aamland, & Hartman, 2015; Morton, Elliott, Cleland, Deary, & Burton, 2017). This is similar to the Pain Science Education approach developed by Moseley and colleagues (Leake et al., 2023). Placing explanation within a biological science of symptoms may also help to move from the cartesian dualism of mind and body towards a more embodied approach to understanding persistent symptoms (Slatman, 2018).

### 1.3 Biographical disruption and repair in persistent physical symptoms

Biographical disruption has been extensively described in people with persistent physical symptoms and clinical syndromes predominantly characterised by symptoms. This includes chronic pain (Toye et al., 2014; Wasson, 2018; Zheng et al., 2013), fatigue (Whitehead, 2006, Asbring, 2001) and multiple symptoms (Spillmann et al., 2017; Nettleton, 2006; Nettleton et al., 2005). The construct of “medically unexplained symptoms” is particularly problematic because the lack of a medical explanation (or in some cases the imposition of an unwanted psychological one) implies that the symptoms may not indicate a medical condition, but rather a problem with the person, or the self (Hartog et al., 2020; Rossen et al., 2019; Werner, Isaksen, & Malterud, 2004). This has been described as an epistemic incongruence (the patient knows the illness in their body, but there is no corresponding illness in the clinician's diagnostic lexicon) (Johansen & Risor, 2016a). In turn this lack of common language leads both to epistemic injustice (Kidd & Carel, 2017) and to the dynamic summed up by Hadler as ‘if you have to prove you are ill, you can't get well’ (Hadler, 1996). An illness narrative which lacks the structure and anchor points of specific findings or diagnoses is commonly – using Frank's typology (1995) – ‘chaotic’ (Nettleton et al., 2005; Frank, 1995). This unstructured, anxiety-inducing chaotic narrative (or ‘anti-narrative’) is in direct contrast to Frank's other two types of illness narrative, the restitution narrative and the quest narrative, both of which provide a hopeful story of meaning and recovery. While biographical disruption could give rise to any of these three narrative types, the chaotic one, observed in people with persistent physical symptoms, is hardest to own, to hear, and to engage with.

While there is an extensive literature on biographical disruption, there is far less about biographical repair in relation to persistent physical symptoms. Patients are involved in a struggle to make sense of their illness experience (Lidén et al., 2015; Rossen et al., 2019). Peer support may act as an impetus to an ongoing process of reconstruction of identity, illness acceptance and coping (Sallinen et al., 2011). This and other socially mediated forms of repair, however, generally happen despite rather than because of medical intervention.

In this paper we propose that providing scientifically plausible (Van Den Bergh et al., 2017) and acceptable (Davies et al., 2020; Salmon, 2007) explanations for symptoms in the context of an especially designed clinical intervention can be a catalyst for biographical repair. Offering and negotiating explanations for symptoms opens a communicative space in which epistemic incongruence in the clinic can be resolved (Den Boeft et al., 2017; Hyland et al., 2016). Explanations ensure medical legitimation and help the patient make sense of the symptoms, both in relation to their body and in the context of their lives. In so doing, they facilitate a shift of focus from the search for causes to acceptance and adaptation through action. In what follows we examine this repair work as it occurs in the process of clinical communication, in the context of a novel intervention for patients with persistent physical symptoms.

### 1.4 A ‘symptoms clinic’ for persistent physical symptoms

In response to the challenge of overcoming the epistemic incongruence around persistent physical symptoms, we have developed a framework for constructing explanations (Morton, Elliott, Cleland, Deary, & Burton, 2017) and also a teachable extended consultation model (Morton et al., 2016a) based on four components: (1) recognition and validation of the person and their illness as legitimate, (2) explanation, (3) action to manage symptoms (based on the explanation) and (4) learning, for both patient and clinician. These have been developed through preliminary studies (Burton, Weller, Worth, Marsden, & Sharpe, 2012; Morton et al., 2016b) and have recently been evaluated in a large multicentre randomised controlled trial with embedded process evaluation (Multiple Symptoms Study 3, abbreviated to MSS3) (Mooney et al., 2022; Fryer et al., 2023).

In this paper we examine how participants in the MSS3 study moved from biographical disruption to features of biographical repair, and the central role that explanation played in this.

## 2 Materials and methods

### 2.1 Study setting

The MSS3 study is a large randomised controlled trial of extended-role GP consultations for patients with persistent physical symptoms (Mooney et al., 2022). The trial contains an embedded qualitative study with two sources of data: consultation transcripts and interviews with participants in the active treatment arm of the trial. The trial involved specially established clinics, initially in 3 UK cities but subsequently delivered online via a health service video consultation platform. 354 participants were enrolled and randomised in equal proportions to receive the intervention or to continue with usual care. Enrolment took place between December 2018 and December 2021 with an interruption between March and August 2020 because of the Covid-19 pandemic. Trial participants had to have multiple persistent physical symptoms of moderate severity (scores of between 10 and 20 on the PHQ-15 questionnaire) without significant physical disease comorbidity.

**Fig. 1:**
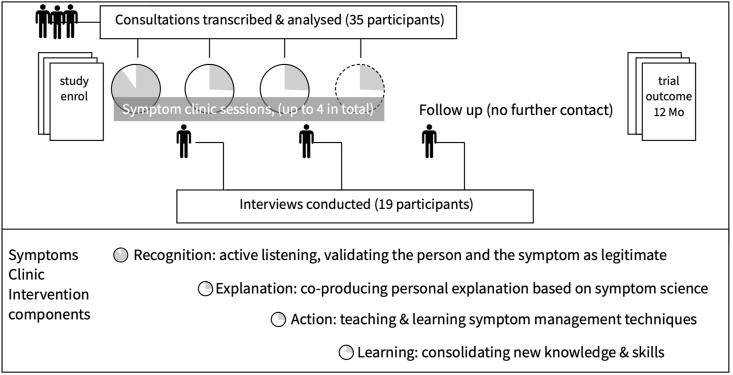
Qualitative study and intervention components.

The intervention comprised a set of 4 extended medical consultations with a specially trained general practitioner (GP). Seven GPs were recruited and trained to deliver the intervention. Training took place over 13 half-day sessions. The first four focused on the content of explanations for persistent physical symptoms, and the next 6 sessions on developing an extended consultation style for the clinics. This used a mix of consultations and 1:1 supervision with a member of the study team. The remaining 3 sessions focused on consolidating skills and knowledge. The study design, intervention content and relationship to the data collection in this study are depicted in Fig. 1.

While the Symptoms Clinic was delivered by specially trained GPs, what it did was very different from ordinary general practice in several regards. First it was provided to patients registered with any participating general practice rather than to patients in the doctor's own practice. Second, consultations were intended to be much longer than normal (approximately 50 min for the first and 15–20 min for up to 3 subsequent consultations). Usually, consultations took place at two-week intervals, but longer intervals were allowed according to circumstances. The aim of the consultations was to take a detailed clinical history, offer and negotiate a diagnostic formulation in terms of body and brain processes which could be used to explain the persistent symptoms, and explore possible self-management interventions. This was summed up in the treatment model as Recognition, Explanation, Action and Learning (Fryer et al., 2023; Mooney et al., 2022). As in conventional GP consultations, the practitioner was encouraged to be flexible within the sessions and to adapt the intervention to each patient rather than to deliver a fixed package of therapy. Clinicians were taught to use explanations based on a contemporary neuroscientific account of symptoms (Henningsen et al., 2018) including central sensitisation (Latremoliere & Woolf, 2009) and in ways broadly comparable to pain neuroscience education (Wood & Hendrick, 2019). These explanations emphasised the reality of symptoms as embodied experiences and also as the consequence of linked brain and body processes. The explanations were adapted to be personally relevant by including elements from the patient's account and offering an interpretation of these experiences through the layered biomedical and psychosocial components of the explanation. Thus, the patient's story was scientifically necessary for the explanation to make sense (and not just elicited for the benefit of being ‘listened to’ prior to having a standardised mechanistic explanation imposed on them).

Importantly, the clinic format was deliberately very different from ‘normal’ general practice. Not only was there more space (time) for the patient to give an account of their illness and articulate their understanding of the problem, but the doctor explicitly co-produced (or at least negotiated rather than prescribed) the explanation and plan for action (Den Boeft et al., 2017; Fryer et al., 2023). Additional features which reduced systemic power differences between the doctor and patient voices included starting the first consultation from a blank canvas and leaving the patient's medical record (ie technologically-and system-mediated account of the patient's history) out of the picture. The primary outcome of the trial was persistent physical symptoms judged by the PHQ-15 self-report scale (Kroenke et al., 2002) at one year after enrolment. The trial results have been submitted elsewhere for publication.

Overall study participants had a median age of 47 (range 18–70). 82% were female and 93% were white and/or had English as their first language. 39% had no formal qualifications after age 16 while 34% had a university degree. We assessed health literacy using the HLS EU-6 survey: at baseline only 36% of participants met the cut-off for “sufficient” health literacy, 49.3% had scores in the range suggesting “problematic” health literacy and 14.7% had “inadequate” health literacy. Patients all had multiple physical symptoms affecting them on a regular basis with the most common symptom groups being fatigue and headache, musculoskeletal pain and gastrointestinal symptoms. Most participants had symptoms in all three groups. The GPs delivering the intervention had between 5 and 25 years of experience. They were recruited by advertisement and undoubtedly did have an interest in the approach of the study. Two had previously led initiatives on pain management and one had extensive counselling/coaching experience in addition to being a GP. However, none of the clinic GPs had worked with the investigators before and all found both the structure and content of the intervention both new and initially challenging.

Ethics approval was granted by Greater Manchester Central Research Ethics Committee (reference 18/NW/0422) on June 25, 2018. All participants provided informed consent, including for the use of consultation recordings.

### 2.2 Embedded qualitative study

The clinical trial included an embedded qualitative study. This had two aims; the first was to evaluate the trial processes, including fidelity to the intervention and understanding the experience of the patients and those delivering the intervention. The second aim was to increase our understanding of the communication and processes of change at play within the intervention. The evaluation of trial processes including fidelity have been published elsewhere (Fryer et al., 2023).

### 2.3 Data collection

Qualitative data was collected in two ways: audio recording of consultations and semi-structured interviews with GPs and patients. All consultations (both face to face and online) with participants allocated to the Symptoms Clinic intervention (N = 176) were recorded using encrypted devices. Approximately one third of consultations were transcribed for quality assurance and analysis, and of these a sample of 35 were used for in-depth qualitative analysis. Selection of recordings for transcription prioritised early patients for each of the GPs delivering the intervention to provide quality assurance and allow any issues to be addressed in supervision.

Interviews were conducted with 19 participants in the Symptoms Clinic arm of the trial. Interviews were conducted either face to face or by phone by one researcher. These were separate from the clinic consultations and were scheduled so that some patients were interviewed shortly after the first appointment while others were interviewed at a later date later in the intervention or after it had been completed. The interviewer had no access to the consultation recordings of interviewees either at the time of the interview or later during analysis (i.e. no patient was included in both the analysis of consultations and the interviews). Interviews lasted between 12 and 46 min and used a semi-structured approach with a topic guide. The topic guide was allowed to evolve over the course of the interviews. Participants for interview were selected purposively, considering main symptom type (pain or fatigue) and employment status. To determine the number of interviews and consultations needed to meet the aims of the study we were guided by ‘Information Power’, in which the expected heterogeneity of data informed the number of interviews (Malterud et al., 2016). Considering the clear aims of the study, the heterogeneity of symptom types, our use of established theory to understand the data, the quality of dialogue (particularly considering the natural dialogue between GP and patient), and the use of a phenomenologically informed analysis strategy, our sample size was both large enough to present a range of experiences and compact enough to establish patterns within the data.

### 2.4 Analysis

We used an inductive thematic approach to analysis of clinic consultations and participant interviews, drawing on sensitising concepts around experience, language, culture, and stories, in order to maximise engagement with this varied dataset. Initial codes were developed and then aggregated into themes in an iterative process by the qualitative researcher. They were then discussed in regular analysis meetings with three other members of the team: a GP and two sociologists. Initial meetings focussed on discussion of singular cases (consultations, transcripts, or interviews) while subsequent meetings moved on to reviewing key themes. Analysis meetings enhanced reflexivity as the team were able to challenge each other's interpretations and reflect upon their own position in relation to the data. Transcripts were coded (using Nvivo-12) according to a number of themes and categories to identify the variation in responses; these were subsequently explored in relation to the literature. Typologies were developed to examine the data as they related to each other within the context of the entire dataset, so that the relationship between themes could be mapped using a visual display of interconnected topics and coded text, which provided a visual representation of the data. Data analysis was conducted using the constant comparative method (Strauss & Corbin, 1990) where data were compared systematically. Emergent themes were analysed iteratively through subsequent interviews to aid conceptual development and assess their variation and consistency across multiple cases. Descriptive accounts were written and examined to assess similarities and differences across the interviews and within single interviews. These accounts offered an overview of each interview so that each case could be analysed in depth. We did not initially conceptualise our data in terms of biographical repair, but as the struggles expressed by patients to explain and accommodate their illness experiences. Biographical repair became apparent during close analysis of the data and regular team discussions, where it became evident that many patients, despite experiencing illness disruption, had moved towards a phase of reconciliation and renegotiation of their disruptive illness state. At this point we began examining the data as a process of biographical/narrative introspection, recalibration and repair.

## 3 Results

In these findings, we first report how patients' persistent physical symptoms resulted in a state of biographical disruption. The unexplained nature of patients’ persistent physical symptoms acted as a block to biographical repair. The lack of explanation of symptoms left individuals constantly negotiating (with themselves or others) whether symptoms represented illness or a problem with themselves as a person. Explanations arrived at in the clinic served to frame symptoms as legitimate and tractable entities. In turn, this facilitated a shift of focus from the self as the problem, to the self as the person living with and adapting to the problem. As such we argue that this represents the unblocking of biographical repair.

### 3.1 Disrupted biographies

As expected, given previous studies of persistent physical symptoms, the presence of symptoms resulted in tangible disruption to physical function, sense of normality, social, and work life. Patients used emotion-laden phrases such as ‘I'm not me anymore’, or ‘taking my life away’, and these phrases were usually found adjacent to descriptions about not being able to do what they want to do. This is in keeping with the sociological research on biographical disruption, which documents people's experiences as characterised by a significant impact on self and identity.

Oh aye, when the pain gets too much, I can’t do anything, it’s taking my life away, I’ve always been active, always went to the gym and it’s just, some weeks it just takes your life over and you think I cannot be doing with this, I can’t, I can’t have something ruin my life and that’s what it does. (G04010 Consultation 1)

Patients described frustration at a lack of diagnosis from previous clinical interactions with primary and secondary care professionals. The lack of explanation for their illness reinforced their struggles, leaving them at a loss in terms of how to manage their symptoms or accommodate them into their lives. With no explicit diagnosis or explanation to work with, participants often persisted with workplace and social activities despite finding them physically difficult. This served to maintain the person's legitimacy and moral worth. While it was often described in terms of the perceptions of others, such as avoiding people thinking ‘she's putting it on’, it was also clear that the perceptions of others were readily internalised:

One of the other big things is also getting friends and family and work to understand what's wrong with you [mmm]. You feel, I’ve felt many times that I'm just a fraud [mmm] and that I'm just er, work shy, lazy, hypochondriac (S10 048).

The lack of explanation meant that, in addition to the work of living with their illness, patients felt they had to work hard at being credibly ill in the eyes of others so as to avoid stigmatisation, or the spoiling of their identity (Goffman, 1959). Part of this work to retain coherence and credibility involved an ongoing effort to make sense of the symptoms. Patients searched for explanations based on scientific research (using online or other media sources), and sought to link these to elements of their own biography in order to “at least create a bit of meaning”:

Because there's no treatment you seem to spend a lot of time researching it and you know, because you're looking for an answer to make you better and so you do come across things and think, that could be relevant, that could be because I had amalgam fillings or because I had an accident… and they may or may not be true but they at least create a bit of a meaning [yeah] for you, you know? (S19054 Interview)

In the absence of treatment, the search for explanations and meaning became the primary form of action through which symptoms could be addressed, without however reaching a satisfactory resolution. As Nettleton (2006) has argued, the reported loss of identity, the struggle to legitimate their symptoms as real, and engaging in a search to acquire some meaning for their illness can be interpreted as features of a ‘chaos narrative’. The accounts of illness in consultations and interviews certainly had these features. In his original formulation of the ‘chaos narrative’ concept, Arthur Frank also pointed to its implications for clinical practice: ‘[t]he worst thing that medical staff can do to someone in the chaos story is to rush him to move on … attempting to push the person out of [the situation] only denies what is being experienced and compounds the chaos’ (1995: 110).

### 3.2 Explanations in the clinic

The intervention specifically addressed key issues relating to biographical disruption. It began with validation of the individual and their experience, offered and negotiated explanations for symptoms, and then explored ways of reducing or adapting to the symptoms. Explanations were co-constructed in a dialogical process where elements of the patient's experience were discussed alongside ideas from contemporary science (Henningsen et al., 2018; Moseley & Butler, 2015; Latremoliere & Woolf, 2009). The multi-layered model of persistent physical symptoms, which clinicians were trained to translate into everyday language, allowed symptoms to be described as embodied experiences that are at least partly explicable by biology of the body and/or brain, and as such are a legitimate medical concern:

Doctor: And what we think happens in patients like that and it’s really common for it to happen is that the problem isn’t in the tissues, the problem is in the processing in the spine and also you’ve got a kind of processor here [yeah] in the brain [ok] below the sub, below the conscious it isn’t something you’re thinking [yeah] this is something that’s happening to the nerves [yeah] and there’s one theory that it’s kind of, this little, these processors get kind of overloaded and this often happens after people have had an operation or injury (GP in Consultation 2 with G01042)

In this example, the focus of the medical explanation is on the “processing” of the neural networks, rather than on a specific pathology. The explanation is offered in a conversational style which encourages the doctor and patient to think together. Acknowledgements from the patient (“yeah”, “ok”) indicate convergent thinking that contrasts with difficulties in communication reported by patients’ (with PPS) in previous studies (Houwen et al., 2019).

Explanations commonly drew on models of the autonomic nervous system with its contrasting components of sympathetic (fight or flight) and parasympathetic (rest and digest) systems. The conversation often involved a reference to mechanisms underlying the symptoms; in the quotation below, the activated sympathetic nervous system “in overdrive” was the mechanism. This state of activation was linked to past events (when a ‘fight or flight’ response might have been appropriate and normal) rather than current thoughts or behaviours. The idea of “overdrive” conveys the notion that the nervous system has become stuck in a response that is no longer relevant to the situation in the present. Thus, the activated sympathetic nervous system becomes the problem to be addressed:

Doctor: It would be completely understandable and logical for your sort of erm sympathetic nervous system to really be in overdrive because of all of that [yeah]. Erm and I think that it, it might be worth considering [mm] whether if that’s true and if that sort of seems to make sense to you whether it might be worth thinking about ways of activating the other bit, the parasympathetic nervous system [yeah], just to counterbalance some of that stuff [yeah] (S13005 Consultation 1)

Explanations explicitly located the reality of symptoms within the (lived) body (Slatman, 2018), as something whose occurrence made logical sense in the context of events or circumstances in the patient’s biography. In this way, they provided the elements for narrative repair.

Explanations also provided a logical bridge to suggesting practical and meaningful actions which are known to modulate the autonomic nervous system, such as slow-paced breathing (Sevoz-Couche & Laborde, 2022) and relaxation:

Doctor: The other things that we can do is because this is all set up on the fight or flight thing, you know there’s danger, we need to be alert all the time [yeah yeah], something that we can do is try and break that cycle by convincing the brain that actually you’re more relaxed, see what I mean? So and that would be…either using relaxation techniques or breathing techniques (GPxx in 2^nd^ consultation with S20002)

This quotation illustrates how the specific *type* of explanation developed in the clinic – and not merely the fact of having an explanation – produced a significant shift in how agency is envisaged in relation to symptoms. In a commonly heard account of symptoms (‘you did too much/were ill … your body needed to stop … your body has now recovered … but you have become deconditioned/afraid to push yourself’) responsibility for the ongoing state and for recovery is attributed squarely to the person. By contrast, in this example the ongoing state is attributed to the brain, and the brain itself is presented as a separate agent endowed with a mind of its own (one the patient has to try to “convince”). This renders the patient as a complex, embodied subject, one internally comprising multiple forms of agency, rhythms, and temporalities, which may be in tension or contradiction with each other and which may need to be harmonised. ‘Brain talk’ in this instance does not imply a form of reductionism; on the contrary, it points to the complex nature of the illness by interweaving layers of neurobiological, psychological, and social explanation. Moving between layers of explanation removes blame, allowing the patient to make sense of their experience without feeling they are personally at fault for their condition. At the same time, it restores a sense of agency, or the feeling that they can do something about the illness.

It is important to stress that explanations based on biological/neuroscientific models were offered in a context of dialogical co-construction as a more general process for making sense of symptoms – rather than as the sole or ultimate truth. Patients contributed to this process by reporting on their own lived experience. The intervention presented them with a way forward based on the current state of their body or brain, but also acknowledged more upstream causes such as past adverse events or challenging current circumstances. In this process of co-construction, the patient had the opportunity to narrate their story, and for their symptoms to be taken seriously, in a clinical setting, as part of that story. The persuasiveness or effectiveness of brain-based explanations as a path to action should not be abstracted from this more general process. Hence, in the example below, the explanation offered by the GP was not “all together new” – the patient had come across brain-based explanations before – but the (new) context of the intervention enabled them to see their symptoms in “a different light”:

It wasn’t all together new, what it was, was just casting a different light on it, so instead of seeing them as a problem it was trying to explain what I could do with the symptoms (S10004 Interview)

Instead of drawing patients into a discourse that sharply differentiates between body and mind, forcing a choice between them, the explanations provided a credible new perspective based on other contrasts, such as the contrast between the past and the present. By focusing on the present, explanations created a space for patients to explore possibilities and rediscover the self:

There’s no miraculous cure, I will give up looking for one so if something works and we can see that it’s making you feel better on those days, then continue with it, there’s no kind of, and I guess my fibromyalgia is different from other peoples as well, so it’s quite individualised. So you really need to monitor what you can and can’t do, and you know, if it makes you feel better then do more of it. So I think that’s the key, the key things that I, that I’ve learnt (G03002 Consultation 4)

### 3.3 When explanation had little impact

While most study participants reported the process of recognition, explanation, action and learning as helpful this was not always the case. In particular, when a doctor proposed explanation or action without first showing sufficient understanding of the patient's lived experiences, patients either became defensive or did not complete the intervention (Fryer et al., 2023).

In some cases, the explanation was accepted but not seen as appropriate. For instance, an explanation of widespread pain sensitisation and autonomic nervous system processes was accepted as plausible but did not address the main trigger which was localised and specific:

Fibromyalgia is like a huge, …a general thing. You know? I’ve really got bad really arthritis. Yeah, I know that. But it’s a different pain altogether. You know? If I reach up, you know, to do something, put clothes on, I can feel it pulling on that specific area. Why doesn’t it do it on that side? (S 14 44)

This type of partial acceptance, characterised by acceptance of the principle but rejection in terms of the individual, is similar to one of the responses observed in a conversation analytic study of psychosomatic attributions (Burbaum et al., 2010) and also in another study of responses to explanations similar to those in the Symptoms Clinic (Den Boeft et al., 2017).

Other patients still sought a definitive diagnosis and cure. Thus, while recognising that acceptance and self-management may have a role, some were not ready to make that step and gave powerful reasons to justify that position:

Where you’re trying to manage the pain in everyday living and everything. This is not, …. ’cause obviously I’ve still got an issue and that’s what he was saying. You know? I’m fighting it. I’m in, basically in denial, you know, and I’m not accepting it. I’m not gonna accept it because I’ve got a family to look after and if I end up in hospital or really, really serious. Cause when I go to hospital I end up in about three or four days. It’s not just like 24 hours. It’s like three or four days (S 22 019)

In both these instances the patient remained trapped in the position of having no explanation and with no openings for a possible biographical repair. Finally, some claimed that as they had lived with persistent symptoms for a long time they have accommodated them within their lives, suggesting a type of ‘biographical continuity’. The symptoms (along with their syndrome diagnosis) had become an integral part of their biography.

I: Has it made things any better at all?

P: A little bit. Yeah I mean, I’ve like I say, I’ve had it that long you just learn to live with it, if you know what I mean. (Laughs) [Yeah, yeah] You see I’ve got two things going on mainly, which is Fibromyalgia which like I say, I’ve had that for years, but I’ve also got sleep apnoea and that’s the biggest problem I’ve got at the minute (S 23 001).

### 3.4 Biographical repair

In the fourth and final consultation, participants were asked to reflect on what had been helpful for them and what they were going to do differently going forward (the learning element of the treatment model). Many described a new level of acceptance and new possibilities for action afforded by the explanation:

I would imagine anybody with this type of condition, always thinks that they’ve either got something else, or there’s something that’s causing it. And the more you read, and the more you look at things on the Internet and that, the, there are all kinds of explanations for these conditions…but actually this is what you’ve got and there’s no magic pill I can take. And that takes time [it does]. (Patient G03002 with GPxx in Consultation 4)

In this instance, as with most other participants, the explanation of symptom mechanisms provided by the doctor did not mean patients were then able to point to a specific, concrete cause to which all symptoms could be attributed. This contrasts with the type of “closure” described by Rossen et al. (2019) in a patient with persistent physical symptoms who was subsequently diagnosed with multiple sclerosis. Rather, in our study, it appears the explanation functioned by providing legitimation for the illness and the assurance that symptoms were being taken seriously; this was sufficient to allow the patient to *move on* from looking for alternative, or definitive, causes.

When patients referred back to the explanation in conversations with the GP, their focus was often on what these implied in terms of actions, rather than seeking more specific details about causal mechanisms. ‘Repair’ in this scenario involved a recalibration of the balance between seeking a cause (looking backwards) or focusing on what they could do (looking forwards) on their journey towards some kind of adjustment to, or acceptance of, their symptoms:

For me it's been useful to learn about the nerves and things as well. But I just feel like I need, not let it kind of control my life really. It's hard because sometimes when you’re in that much pain. But I think it's been helpful, just you explaining not to do too much and trying to build it up gradually. (G03017 with GPxx in 4^th^ consultation)

Engaging in repair work facilitated by the explanations could be difficult, however, in so far as it implied having to let go of narratives and actions previously regarded as helpful coping techniques. In our next excerpt, the patient describes the realisation that their previous approach to their illness – as something temporary, that could be cured by “just having a few weeks off work” and “just chilling out” – had in fact been counter-productive:

I think [I’ve learned] a couple of things. Firstly, that I can’t just decide that I’m not going to have it anymore. Also, that that’s not going to work, just having a few weeks off work and being good for little while, do this, just chilling out, isn’t going to cure it, it’s going to come back. There’s limits that you need to manage… and I’m really not good at that. So that’s been hard to accept … and in my normal techniques will actually work against, you know my normal coping techniques will work against what it, what it is. So that’s been hard to accept that I have it (Patient G04002 with GP xx in Consultation 4)

The attitude of this patient (who held the idea that they could “just decide” to not be ill anymore) might be described as a form of denial of the chronic and complex nature of the illness. In a study of patients with malignant brain tumours, Salander et al. (1996) have challenged conventional psychoanalytic interpretations of denial as a negative ‘defence mechanism’, proposing instead that such strategies should be regarded as a form of repair, or ‘reconstructive activity’, through which patients are able to ‘create hope’ (1996: 993). In the situation of patients with persistent physical symptoms, however, this way of creating or maintaining hope can act as an obstacle to acceptance, which appears necessary for a more lasting and fundamental form of repair to take place. The excerpt above clearly illustrates the *work* involved in achieving acceptance. This work is “hard”, and perhaps counter-intuitive, because it requires letting go of familiar strategies of repair, or what the patient had hitherto imagined might restore their normality. The explanation allowed them to understand how their “normal techniques” could actually “work against” them, facilitating a re-orientation of their perspective towards the possibility of a “new normality” (Locock et al., 2009) as a form of repair.

While some patients’ symptoms did improve, others saw little change and yet still perceived a benefit from the intervention:

I can definitely see the benefits of it (slow paced breathing), just in, well just day to day life in general, cos I could feel a difference in myself by doing it. Pain wise I couldn’t really tell one way or another… it didn’t make a huge difference in the pain but it did, I did feel better as a result of doing it. (P3 in Consultation 3 with GP1)

While on one level this might be read as suggesting there was simply a generic benefit of body-regulating activities such as slow-paced breathing, for most patients it was necessary to go through the explanation phase to get to this action. The following quote shows how engaging with the person's uncertainty in a culturally acceptable way (using science for a “sciencey person”) was important in helping the patient commit to the self-management actions they had discussed:

If I’d gone in for like a normal ten-minute appointment and my GP had said try mindfulness, I would’ve said yeah OK, just refill my prescription please…the way that you’d explained it with medical evidence as well, cos I’m a sciencey person, has been really helpful, so it’s made me want to commit to it (S17068 Interview)

Some individuals reflected on the intervention using terms that are explicitly evocative of a process of repair, as in this example:

It suddenly made me think of myself as much more whole than even I had imagined (S10004 Interview).

## 4 Conclusions

People with persistent physical symptoms, particularly where there is no adequate underlying diagnosis, experience biographical disruption which impacts upon their sense of self. We found that a clinical intervention specifically designed for patients with these conditions can facilitate a process of biographical repair. This repair is characterised by elements of both acceptance and agency, finding a new normal, and coming to terms with a revised biography which incorporates elements of the illness without being defined by it. What is new in this paper is that we describe the process happening over a short period of four extended medical consultations and demonstrate the centrality of providing an explanation of symptoms based on recent scientific research.

In this discussion we consider our findings in the context of existing interventions for persistent physical symptoms and other evidence about the role of explanation in “unexplained” symptoms; links between the biographical approach and other narrative approaches, particularly Frank's narrative typology; and the relationship between biographical repair and living well.

### 4.1 Interventions for persistent physical symptoms

There have been relatively few interventions for persistent physical symptoms (or “medically unexplained symptoms”) in primary care and two recent reviews found no clear evidence of effectiveness (Byrne et al., 2022; Leaviss et al., 2020). In specialist settings, psychological therapies such as cognitive behavioural therapy (CBT) have moderate effects (Kleinstauber et al., 2011), but these therapies are commonly not available and have limited acceptability to patients. While the Symptoms Clinic is less explicitly psychological, it also draws on aspects of CBT and many of its components map to desirable intervention characteristics in a recent realist review (Leaviss et al., 2020). The Symptoms Clinic, shown to be effective in the clinical trial (accepted for publication), provides an important opportunity for intervention in a significant group of patients for whom little is currently available.

### 4.2 The role of explanation

Following a series of in-depth studies of clinical consultations, Salmon proposed the need for a curriculum of explanation for persistent symptoms (Salmon, 2007) as a necessary step for helping people with ‘unexplained’ symptoms. While many of the recommendations he made were influential in the design of the Symptoms Clinic, two points are particularly important. The first is a reminder of the risk, in using a biomedically informed explanation, that symptoms are reified as something concrete and wholly detached from the person. This has been challenged as a disavowal of the psychosocial (Greco, 2012). However, our analysis found little to suggest that happening in this study. Rather, explanations were co-constructed as multi-layered accounts in which symptoms appear as embodied (Slatman, 2018; Kirmayer & Gómez-Carrillo, 2019). The process of co-construction enacted a form of speculative pragmatism, where explanations are tested not by whether they are true in terms of their ability to accurately describe causal mechanisms or predict the future (outcomes), but by whether they are useful in facilitating an opening towards new actions and possibilities (Greco, 2017). The second point, echoing Kirmayer ((1994), was the necessity that a curriculum of explanation should “address how practitioners balance the authority that is needed to set out an explanatory framework with a partnership to ensure that the explanation that is co-constructed is meaningful to the patient and practitioner”. Again, the analysis suggests that this balance of authority was indeed critical, and where balance was not achieved, the intervention failed. While we have argued that explanations are essential for bridging the epistemic gap between doctor and patient (Johansen & Risor, 2016b), explanations presented without the personalised context in this study have little effect (Weigel et al., 2023). Importantly, for individuals whose illness narratives can be recognised as chaotic (Nettleton, 2006) the explanations were introduced in a way which “honoured the chaos” (Frank 1995: 110).

A short term enhanced medical communication intervention, centred around hearing and validating the patient's illness narrative and making sense of it with multi-layered explanations, enabled people with multiple persistent physical symptoms to reframe their symptoms in a way that enabled them to *act* differently and learn a new balance between acceptance and agency; regain some control over their symptoms and move to a new normality which had meaningful continuity with their life prior to disruption. This can be understood as a process of biographical repair.

## Funding

This study is funded by the NIHR Health Services and Delivery Research programme (project: 15/136/07). The views expressed in this publication are those of the authors and not necessarily those of the NHS, the NIHR or the Department of Health.

## CRediT authorship contribution statement

**Tom Sanders:** Writing – review & editing, Writing – original draft, Supervision, Funding acquisition, Formal analysis, Conceptualization. **Kate Fryer:** Writing – review & editing, Writing – original draft, Investigation, Formal analysis, Data curation, Conceptualization. **Monica Greco:** Writing – review & editing, Writing – original draft, Funding acquisition, Formal analysis, Conceptualization. **Cara Mooney:** Writing – review & editing, Writing – original draft, Funding acquisition, Formal analysis, Data curation. **Vincent Deary:** Writing – review & editing, Writing – original draft, Funding acquisition, Formal analysis, Conceptualization. **Christopher Burton:** Writing – review & editing, Writing – original draft, Methodology, Investigation, Funding acquisition, Formal analysis, Conceptualization.

## Declaration of competing interest

The authors declare that they have no known competing financial interests or personal relationships that could have appeared to influence the work reported in this paper.
